# Innate Immunity Activation and RNAi Interplay in Citrus Exocortis Viroid—Tomato Pathosystem

**DOI:** 10.3390/v10110587

**Published:** 2018-10-26

**Authors:** Thibaut Olivier, Claude Bragard

**Affiliations:** 1 Life Sciences Department, Walloon agricultural research Centre, Rue de Liroux 4, 5030 Gembloux, Belgium; 2 UCLouvain, Earth&Life Institute, Croix du Sud 2bte L7.05.03, 1348 Louvain-la-Neuve, Belgium; claude.Bragard@uclouvain.be

**Keywords:** pospiviroid, innate immunity, degradome, transcriptome, RNA silencing, Dicer-like, argonaute, CEVd, systemic acquired resistance, SAR

## Abstract

Although viroids are the smallest and simplest plant pathogens known, the molecular mechanisms underlying their pathogenesis remain unclear. To unravel these mechanisms, a dual approach was implemented consisting of *in silico* identification of potential tomato silencing targets of pospiviroids, and the experimental validation of these targets through the sequencing of small RNAs and RNA ends extracted from tomatoes infected with a severe isolate of *Citrus exocortis viroid* (CEVd). The generated RNA ends were also used to monitor the differentially-expressed genes. These analyses showed that when CEVd symptoms are well established: (i) CEVd are degraded by at least three Dicer-like (DCL) proteins and possibly by RNA-induced silencing complex (RISC), (ii) five different mRNAs are partially degraded through post-transcriptional gene silencing (PTGS), including argonaute 2a, which is further degraded in phasiRNAs, (iii) Dicer-like 2b and 2d are both upregulated and degraded in phasiRNAs, and (iv) CEVd infection induced a significant shift in gene expression allowing to explain the usual symptoms of pospiviroids on tomato and to demonstrate the constant activation of host innate immunity and systemic acquired resistance (SAR) by these pathogenic RNAs. Finally, based on *in silico* analysis, potential immunity receptor candidates of viroid-derived RNAs are suggested.

## 1. Introduction

Viroids are the smallest known plant pathogens consisting of small single-stranded and circular RNA molecules [1]. Although viroids differ from most RNA viruses by the fact that they do not code for any protein, replicate in the nucleus or in the chloroplast, and form imperfect double-stranded RNA (ds-RNA) structures, like RNA viruses, viroids are able to activate and resist RNA silencing [2], also termed RNA interference (RNAi), activate systemic acquired resistance (SAR) [3], and probably innate immunity [4]. Recently, the ability of plants to recognize viral ds-RNAs as pathogen-associated molecular patterns (PAMPs) and mediate PAMP-triggered immunity (PTI) has been demonstrated [5].

In light of these discoveries, viroids thus offer an opportunity to: (i) better understand how RNA silencing works, (ii) figure out if plant defenses possess pattern recognition receptors (PRRs) or disease resistance proteins which are able to recognize RNAs as PAMPs or effectors and trigger PTI or effector-triggered immunity (ETI) respectively [6], (iii) comprehend how viroids can elicit SAR [7], and (iv) unravel the possible interplay between these three major plant defense systems.

Besides these unanswered questions related to defense strategies, the underlying molecular mechanisms leading to viroid symptom induction and modulation are also still largely unknown, despite more than 30 years of research efforts. Currently, three main theories—which are potentially interconnected—are put forward to explain how pospiviroids trigger the first reactions: (i) the first hypothesizes interaction between viroids and unknown plant proteins which would initiate symptom development [8], the two others postulate that viroid-derived small interfering RNAs (vd-sRNAs) trigger the modulation of important protein synthesis through (ii) RNA directed DNA methylation (RdDM) [9] or (iii) post transcriptional gene silencing (PTGS) [10].

It is now well established that viroid and virus RNA structures are recognized by host ribonuclease III-like proteins, Dicer-like (DCL), and cleaved into 21–24-nucleotide vd-sRNAs [11,12]. Transcriptional gene silencing relying on sequence complementarity between vd-sRNAs and host genome is thus a possible way through which pospiviroids induce symptoms; this was demonstrated for a variant of *Peach latent mosaic viroid* inducing peach calico symptom [13]. Although several PTGS targets of *Potato spindle tuber viroid* (PSTVd) have been identified, e.g., PlantWD-40 repeat proteins [14], two genes involved in gibberellin or jasmonic acid biosynthesis [15], a chloride channel protein CLC-b-like, and a ribosomal protein S3a-like [16], divergences between studies exist, and the mechanisms leading to symptoms remain elusive.

The potential induction of symptoms through RdDM was experimentally tested by Wassenegger et al. [17] who demonstrated that PSTVd cDNA integrated into the genome of *Nicotiana tabacum* becomes specifically methylated upon PSTVd replication. However, no methylation of non-transgenic plant DNA due to viroid infection was demonstrated, and the RdDM guide molecules seem to be longer than the 24-nt vd-sRNAs [18].

Regarding the protein-viroid interactions, several tomato proteins were found to interact with viroids [19,20,21,22], but although some, like Virp1 [23], was found to be essential for their infectivity, the roles they play in symptom modulation are not trivial. Apart from their interaction with proteins of the RNAi machinery, viroid-derived ds-RNAs could directly bind to PRR/disease resistance proteins and trigger PTI/ETI. In mammals, the protein kinase RNA-activated (PKR) is, for instance, known to bind ds-RNAs and phosphorylate the translation initiation factor eIF2α, which leads to translation initiation repression. Despite early reports of plant PKR (pPKR) activity in virus-infected plants [24,25,26], no specific kinase could be purified, and the sequence of a putative PKR ortholog is absent from plant genomes [27]. However, the recent discovery of a ds-RNA-induced immunity independent of dicer-like proteins [5] offers hope in the search for a plant ds-RNAs receptor.

While it is thought that pospiviroids replicate latently in many solanaceous ornamentals, eight species of the genus [28] replicate and produce symptoms on tomatoes. This suggests that the induction of symptoms would be independent from the replication and trafficking, and would be rather specific to some susceptible species. However, some mild pospiviroid strains are known to induce very few symptoms on the fast-growing tomato, so that it cannot be excluded that the impact of pospiviroids on slow-growing ornamentals might be difficult to highlight. Conversely, tomato lethal strains of pospiviroids have been identified.

In order to understand how plants activate their defenses against viroids, and how these pathogens escape such defenses while inducing symptoms, a pathosystem consisting of tomato plants cv. Heinz 1706 was infected with a severe isolate of *Citrus exocortis viroid* (CEVd), and was studied through the lens of the transcriptome and the degradome using high-throughput sequencing technologies. Basically, a dual approach was implemented consisting of: (i) prior *in silico* identification of potential targets of pospiviroids on tomato coding sequences, (ii) the experimental validation of PTGS and RdDM targets through small RNA and parallel analysis of RNA ends (PARE) sequencing, and (iii) the differential gene expression analysis between infected and mock tomato plants by the quantification of PARE and small RNA reads in all known genes.

These analyses allowed us to (i) highlight new tomato PTGS targets, which unravel both counter defense strategies of pospiviroids and plant defense mechanisms, (ii) further understand the decay pathways of pospviroids, (iii) gain new insights in the transcriptional shift induced by pospiviroids, demonstrating the activation of the innate immunity and SAR by CEVd, and explaining the symptom induction of these pathogens on tomatoes, and (iv) propose potential plant receptors triggering innate immunity and SAR in pospiviroid infected plants.

## 2. Materials and Methods

### 2.1. In Silico Analyses of Artificial Pospiviroid Derived vd-sRNAs

Potential tomato PTGS targets of pospiviroids, as well as the pospiviroid genomic regions involved in these interactions, were found using BWA version 0.7.12 [29]. 21 nt artificial vd-sRNAs were generated from a set of 891 pospiviroids complete genomes: 313 *Potato spindle tuber viroid* (PSTVd); 128 *Chrysanthemum stunt viroid* (CSVd); 253 CEVd; 83 *Columnea latent viroid* (CLVd); 11 *Mexican papita viroid* (MPVd); 58 *Pepper chat fruit viroid* (PCFVd); 28 *Tomato apical stunt viroid* (TASVd); and 11 *Tomato chlorotic dwarf* (TCDVd). These were circularized by copying the first 21 nucleotides at the end of each sequence. The domains to which the artificial vd-sRNAs belonged were computed according to the position of the vd-sRNA central nucleotide in an annotated alignment of the 891 pospiviroids sequences, and based on the domain boundaries identified by Keese and Symons [30]. To allocate the mapping sites to tomato genomic features, intergenic regions, exons and introns, the tomato coding sequences (ITAG3.2), and the feature file “models.gff”, provided by the International Tomato Genome Sequencing Project, were used. Minimum free energies of duplexes were computed according to Serra and Turner [31].

### 2.2. CEVd Inoculation

A *Solanum laxum* isolate of CEVd (12/0288/VI.4) inducing severe stunting, leaf chlorosis, and deformations, as well as leaf necrosis on tomatoes cv. Minibel, was inoculated to tomato plantlets cv. Heinz 1706-BG (*Tomato* Genetics Resource Center accession: LA4345) at the first true-leave stage with a mix of 100 mg of infected or healthy tomato leaves in 2 mL of distilled water. Inoculated plants were grown at 26/24 °C day/night temperatures with a 16 h daylight photoperiod, and pospiviroid infections were checked by RT-PCR [32]. A pair of both healthy and infected plants were selected for RNA extraction and sequencing.

### 2.3. RNA Extractions and Sequencing

The top 5 cm of two infected plants and two healthy plants consisting of leaves, stem, petioles, and apex were sampled 47 days after the inoculation and approximately at the middle of the light phase. The samples were immediately crushed in liquid nitrogen and stored at −80 °C until extraction. Small RNAs and total RNAs were extracted using a Spectrum Plant Total RNA kit (Merck, Darmstadt, Germany), according to the manufacturer’s instructions, for both types of RNAs. Four small RNAs and 4 PARE Illumina libraries were prepared and sequenced on Illumina NextSeq500 by GenXPro GmbH (Frankfurt am Main, Germany). Small RNA libraries were prepared by selecting 18–64 bp long RNAs and using “TrueQuant” adapters. PARE libraries were prepared following a protocol derived from German et al. [33], but with “TrueQuant” adapters, and skipping the MmeI digestion step to allow the selection of longer fragments of uncapped RNAs. Small RNA and PARE reads are available at SRA accession: SRP150214 (Table S1).

### 2.4. In Silico Analyses of Experimentally Generated Sequences

Alignments of small RNA and PARE reads on CEVd were performed using BWA on a newly-constructed CEVd sequence allowing 2 mismatches for small RNAs and alignment seeds of 17 nucleotides for PARE reads.

Potential silencing sites were identified by mapping of CEVd-derived small RNAs on tomato genomes (version SL3.0) supplemented with tomato chloroplast and mitochondrion genome sequences (accession numbers: NC_007898.3 and NC_035963.1 respectively). For this analysis, BWA was used, and 3 mismatches per duplex were allowed. To assess the differential expression of genes, PARE reads were mapped on tomato coding sequences (release ITAG3.2) supplemented with publicly-available tomato miRNAs using BWA and a mapping seed of 17 nucleotides. Mapped reads were quantified in each gene for the four samples using Salmon [34]. Differential gene expression analysis based on read quantification was then carried out using the package Edger [35,36] in R version 3.2.3. Gene ontology enrichment and analysis was performed using the package TopGO [37] in R.

To highlight phasi-RNA activation, microRNA genes differential expression and hot spots of vd-sRNA in genes, small RNAs sequences were mapped on tomato coding sequences (release ITAG3.2) supplemented with publicly-available tomato miRNAs using BWA by allowing a maximum of 2 mismatches. Gene and microRNA-derived small RNAs were quantified for each gene and screened for quantification differences using the same procedure as for PARE reads. PhasiRNA detection was performed using the software UNITAS 1.5.1 [38].

Detection of vd-sRNA triggered PTGS cleavages was performed visually by looking at each potential PTGS site identified by mapping, as described above, for differences of PARE 5′ end frequencies between infected and mock samples. When consistent frequency differences were found, the statistical significance of these differences was assessed according to Poisson regressions using R. Vd-sRNAs involved in the PTGS sites were then identified by considering the cleavage site between the 10th and 11th nucleotides of the corresponding 21 nt long vd-sRNAs.

To report the differences in read quantification, the log to base 2 of the fold change (logFC), i.e., the normalized number of reads in CEVd-infected samples divided by the normalized number of reads in mock samples, was used. The statistical significance of this fold change was expressed using the false discovery rate (FDR).

## 3. Results

### 3.1. Analysis of Tomato PTGS Targets of In Silico Generated Pospiviroid vd-sRNAs

**Figure 1:**
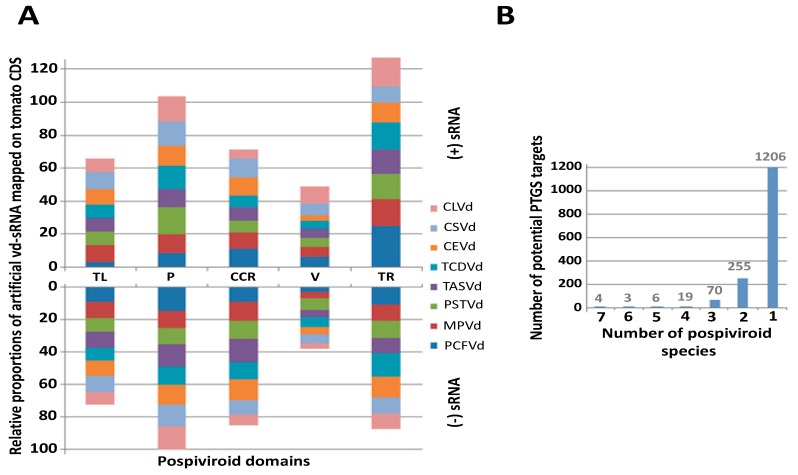
(**A**) Distribution among the five pospiviroid domains of the percentage of artificial 21 nt vd-sRNA mapping to tomatoes CDS for each of the eight pospiviroid species able to infect tomatoes (three mismatches/indels allowed). The upper part of the bar graph shows targets of the positive strand of pospiviroids; the lower part shows targets of the negative strand. Statistical differences between domains are represented as binomial regression groups (*P* < 1e−5 above the upper bar of each domain. The five pospiviroid domains are mentioned according to the following abbreviations: TL—Terminal left; P—Pathogenicity, CCR—Conserved central region; V—Variable; TR—Terminal right. (**B**) The number of potential PTGS targets shared among pospiviroid species according to the mapping of *in silico*-generated vd-sRNAs from a set of 891 complete genome sequences of pospiviroids. Here, only the most thermodynamically-stable sites of Table S2a (ΔG ≤ −25 kcal/mol) were reported in this graph.

Mapping analysis of *in silico*-generated vd-sRNAs on tomato CDS showed that the similarities between pospiviroids and tomatoes are distributed in the five genomic domains of pospiviroids in a similar way for the eight pospiviroid species able to infect tomatoes: TASVd, CLVd, CEVd, PSTVd, PCFVd, CSVd, TCDVd, and MPVd (Figure 1A). Statistical differences were found between each of the five domains in terms of proportions of mapped vd-sRNAs for each of the eight pospiviroid species, and regardless of the strand polarity. The terminal right domain collected the highest number of mapping hits, while the variable domain showed the smallest number (Figure 1A).

Although tomato/pospiviroid similarities are distributed in the five domains in a similar manner for all studied pospiviroid species (Figure 1A), in-depth analysis of these results showed that no potential PTGS target is shared among all eight species, and about three quarters of similarities are only shared between strains of the same species (Figure 1B and Table S2a). Nevertheless, despite this diversity, several gene ontology terms associated with hypothetical target genes were significantly overrepresented, among which terms linked to cytoskeleton (mainly kinesins), kinases, membrane transporters, and 1,3-beta-d-glucan biosynthesis were the most overrepresented (Table S2b highlighted in gray, purple, yellow and orange respectively).

In total, 2303 potential PTGS targets were found to be shared between at least two pospiviroid species: 639 kinases, including many MAP and receptor like kinases (RLKs), 73 RNA-binding proteins, 89 disease resistance proteins, 55 WD40 repeat-like superfamily proteins, and 47 pentatricopeptide repeat-containing proteins (Table S2a). Remarkably, proteins related to RNA silencing were also present in the list, like argonautes (1, 2 and 5), two suppressors of gene silencing 3 (SGS3) involved in PTGS (Solyc05g005450 and Solyc11g066990), as well as one factor of DNA methylation 1 (Solyc03g117040) and one protein INVOLVED IN DE NOVO 2 (Solyc05g054420) involved in RdDM. Of these 2303 gene targets, 309 were found to be upregulated in the infected samples analyzed in this study.

Strikingly, among the four genes found as potential common targets shared between seven pospiviroid species and whose duplexes minimum free energy were lower than −25 kcal/mol, two TIR-NB-LRR genes (Solyc08g005510 and Solyc11g011060) presenting similarity with TMV resistance protein N, and an endoplasmic reticulum-type calcium-transporting ATPase 3 (Solyc07g022790), were highlighted. Similarly, shared between five pospiviroid species, two disease resistance proteins, i.e., a protein homologous of At1g12280 belonging to the NB-LRR family (Solyc02g037540.2.1) and an uncharacterized protein (Solyc05g007170.3.1), were found as additional potential targets. The analysis of secondary structures of the corresponding vd-sRNA/mRNA duplex confirms some of the many interesting potential PTGS targets, since genes related to plant disease, RNA-binding proteins, or transcription factors showed thermodynamically stable duplexes (Table S2a).

In order to validate this strategy of PTGS target discovery, a severe strain of CEVd and the fully-sequenced *Solanum lycopersicum* cv. Heinz 1706-BG, were chosen for the analysis of both viroid and tomato degradomes.

### 3.2. CEVd Degradome and Its Tomato Silencing Targets

The mapping of sequenced-RNAs on the CEVd genome showed that, in agreement with pospiviroid RT-PCR results, CEVd-derived reads were only present in infected samples. The analysis of single nucleotide variants (SNVs) revealed that three CEVd haplotypes, with a maximum of two SNV differences, were present in the infected plants (GenBank accessions: MH476291, MH476292 and MH476293). No other virus or viroid could be detected in the infected and mock-inoculated samples based on BLAST analyses of small RNA reads on a large set of plant viruses and viroids.

**Figure 2:**
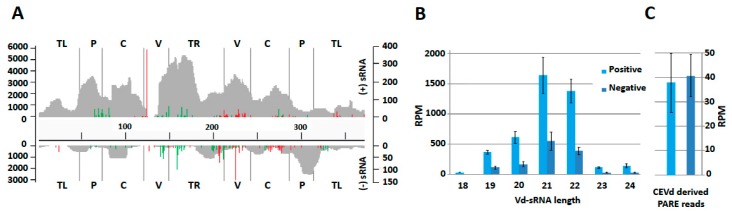
(**A**) Distribution of vd-sRNAs along the CEVd genome (positive strand above and negative strand below) and in the five domains. (18–24 nt). Most frequent 5′ and 3′ ends of RNA ends are depicted in green and red respectively. The left scale refers to vd-sRNA position count, while the right scale refers to the number of 5′ and 3′ RNA ends. The five pospiviroid domains are mentioned according to the following abbreviations: TL—Terminal left; P—Pathogenicity, C—Conserved central region; V—Variable; TR—Terminal right. (**B**) Distribution of vd-sRNA according to their length and polarity. Means of read per million (RPM) are depicted with error bars showing the standard deviations observed between biological replicates. (**C**) Mean abundances (in RPM) of CEVd derived PARE reads for both polarities.

The distribution along the CEVd reference genome of the reads from the two replicates of small RNAs and PARE sequences showed very similar patterns indicating a good repeatability (Figures S1–S3). The number of reads was higher for the positive strand than for the negative strand, and peaks on both strands were clearly visible (Figure 2A). Size distribution of vd-sRNAs showed that 21-nt and 22-nt sRNAs were the most abundant for both polarities (Figure 2B). The comparison of vd-sRNA maps on CEVd genome for each sRNA length (from 18 to 24-nt) showed very similar patterns between biological replicates for both polarities. These similarities were especially pronounced for 21, 22, and 24-nt vd-sRNAs (Figures S1 and S2). While positive strand vd-sRNAs were three times more represented than their negative counterparts (totals of 32,647 and 9519 respectively), remarkably, CEVd-derived RNA ends showed a relatively balanced polarity repartition (Figure 2C).

The numbers of 5′ and 3′ ends of RNA ends for each genomic position were assessed, and the most frequent sites (corresponding to outliers according to Poisson distribution) were plotted on plus and minus vd-sRNA maps (Figure 2A). This analysis showed that several positions along the CEVd genome are overrepresented in terms of 5′ and 3′ extremities of RNA ends.

Unexpectedly, in both polarities, the positions with the most abundant RNA end extremities were those of 3′ RNA ends, both flanking the GC-box of the V domain in opposite strands. Groups of 3′ and 5′ RNA ends hot spots were also clearly visible across the genome in both polarities, indicating preferential ends/cleavages at these sites (see also Figure S3).

**Figure 3:**
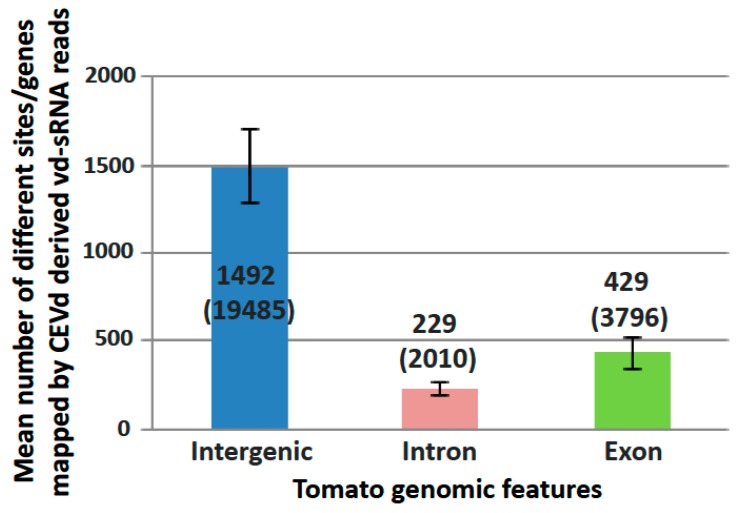
Mean number of different sites/genes mapped by CEVd derived sRNAs for each of the three tomato genomic features: intergenic region, exon (3′ and 5′-UTR included), and intron. Standard deviations between samples are depicted as error bars. Mean number of reads for each feature are shown in parentheses.

The potential tomato targets of gene silencing were identified by the mapping of vd-sRNAs on tomato genome rather than CDS in order to check both RdDM and PTGS potential targets. All vd-sRNA mapping sites on the tomato genome were then clustered into one of the three following genomic features: intergenic regions, exons, and introns (Figure 3).

To assess the possible RdDM induced by CEVd, the differential expression of genes located less than 1 kb from intergenic vd-sRNA mapping sites were checked. The analysis demonstrated that eight genes located less than 1 kb downstream of a vd-sRNA intergenic mapping site were downregulated, as expected in RdDM (Table S3, lines highlighted in green). Impact on expression of gene body methylation is less clear, but many sites in introns and exons could serve as RdDM sites directed by vd-sRNAs.

**Figure 4:**
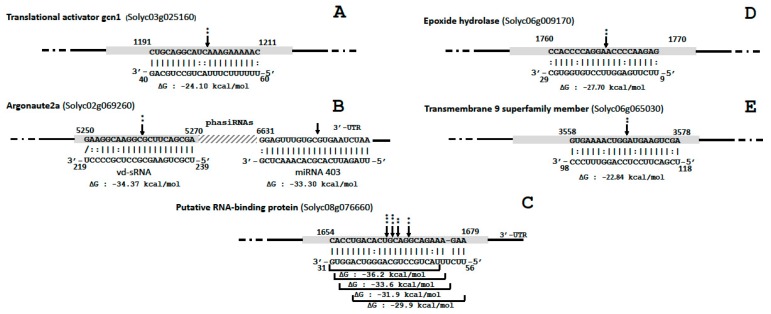
PTGS targets site of CEVd vd-sRNAs of the five genes highlighted in this study: (**A**) translational activator gcn1, (**B**) argonaute 2A, (**C**) putative RNA-binding protein, (**D**) epoxide hydrolase and (**E**) putative RNA-binding protein The arrows indicate the predicted RISC mediated cleavage sites with their respective probability computed according to Poisson’s regression of normalized counts of 5′ RNA ends (* *P* ≤ 0.05; ** *P* ≤ 0.01; *** *P* ≤ 0.001). Complementarity between nucleotide pairs was depicted as follows: | for Watson-Crick base pairs, / for wobble base pairs and: for mismatches. Coding regions of genes are depicted as gray rectangles; intron and 3′-UTR are represented as black lines. The sequences are shown in the complementary polarity. The PairFold online tool was used to predict the minimum free energy (ΔG) of RNA duplexes.

Likewise, possible PTGS targets were evaluated for genes where vd-sRNAs mapped in exons, by assessing the difference in normalized number of 5′ RNA ends in infected samples compared to mocks at expected PTGS sites. Moreover, the differential quantification of small RNAs surrounding the vd-sRNA mapping site was assessed to check the possible production of phased, secondary, small interfering RNAs (phasiRNAs). These analyses showed that five genes, out of the 261 common genes presenting vd-sRNA similarities in both infected samples (Table S4a), were significantly targeted by vd-sRNAs (Figure 4).

The first PTGS target was a gene annotated as a translational activator gcn1 (Solyc03g025160.3.1), for which an accumulation of vd-sRNAs and a significantly higher number of 5′ RNA ends were observed in infected samples compared to mocks (*P* = 0.003) (Figure 4A). Although gcn1 was predicted as a potential PTGS target using pospiviroid artificial vd-sRNA, this gene was not found to be differentially expressed in infected samples according to PARE read quantification. These vd-sRNAs derived from a piece of the negative strand overlapping the upper TL and P domain junction, corresponding to the virulence modulating (VM) region [39]. Pospiviroids artificial vd-RNAs analysis showed that several tested strains of CEVd (14 out of 253 sequences) and all 28 tested strains of TASVd shared this gene target.

The second gene coding for the argonaute 2A (Solyc02g069260.3.1) showed a higher number of 5′ RNA ends at the vd-sRNA mapping site (*P* = 0.019). The CEVd domain involved being a part of the positive strand of the lower V domain. Remarkably, the mapping of small RNAs from infected samples presented a two-hit pathway phasiRNAs profile triggered in 5′ by vd-sRNAs and 1361 nt downstream by the microRNA, miR403 (Figure 4B). However, it should be noted that miR403 was not found to be differentially expressed in infected samples compared to mock, and that both infected and mock samples presented a characteristic degradation profile at the miR403 PTGS site. It is also worth noting that argonaute 2A was the gene for which the difference in terms of mapped small RNAs between infected and mock samples was the highest and the most significant (logFC 11.1, FDR 3.50e−24). This gene was also found to be significantly upregulated according to PARE quantification (logFC 2.7, FDR: 0.0002). Pospiviroids artificial vd-RNAs analysis showed that several tested strains of CEVd (18 out of 253 sequences) and of PCFVd (23 out of 58 sequences) shared this argonaute 2A target site.

A third gene, coding for a putative RNA-binding protein (Solyc08g076660), although not differentially expressed according to RNA ends, was found to present a significantly higher number of PARE read 5′ ends than in mocks (*P* ≤ 0.01) at four positions within the most vd-sRNA targeted locus (Figure 4C). The analysis of small RNA sequences at this locus showed that they all derived from the negative strand of the 5′ part of the VM region covering a region of 26 nt. The gene Solyc08g076660 was also found to collect significantly more small RNAs in infected plants (logFC 6.9, FDR 3.1e−12), primarily due to this vd-sRNAs hot spot. Concerning the function of this CEVd PTGS target, BLASTX analysis of the corresponding mRNA revealed a 100% identity with a protein predicted as a nucleolar protein 12 (GenBank accession: XP_004245924.1), whose exact role is unknown, but which was found to bind to mRNA [40]. Pospiviroids artificial vd-RNAs analysis also showed that this target is shared within almost all tested strains of CEVd (248 out of 253 sequences) and TASVd (27 out of 28 sequences), but is confined to these species.

The fourth PTGS site was an epoxide hydrolase (Solyc06g009170) targeted by vd-sRNAs from the terminal left of CEVd (Figure 4D). *In silico* analysis showed that this site is shared between most of the analyzed strains of TASVd and CEVd.

In the fifth PTGS target, vd-sRNAs from the central conserved region form a duplex with mRNAs of a poorly-characterized transmembrane 9 protein (Solyc06g065030) (Figure 4E). *In silico* analysis showed that this site is shared between many strains of TASVd, CSVd and CEVd.

### 3.3. Tomato Transcriptome Changes upon CEVd Infection

The analysis of the differential expression of RNA ends showed that in CEVd infected plants, 4097 and 3519 genes/ncRNAs were significantly upregulated and downregulated respectively compared to the mock-inoculated plants (|logFC| > 1 and FDR < 0.05) (Table S5). The quantification of small RNAs showed that 67 and 66 genes/ncRNAs collected significantly more and less small RNA respectively in CEVd infected plants (|logFC| > 1 and FDR < 0.05) (Table S6).

According to PARE reads, gene ontology (GO) enrichment analysis revealed that many GO terms were significantly overrepresented in upregulated and downregulated genes identified in infected plants (Tables S7 and S8 respectively).

Because the gene content of the highlighted GO terms greatly overlapped, to avoid redundancy, ontology enriched genes were reported according to the following categories: kinases, pathogenesis-related (PR) genes, transcription factors, oxidation-reduction process, protein ubiquitination, DNA methylation and chromatin remodeling, RNAi, and hormone related genes. Also, when it comes to gene annotation, BLASTX analysis of differentially-expressed genes revealed that several discrepancies arose. BLASTX confirmation was thus performed for some specific genes reported hereafter.

#### 3.3.1. Kinases

The first category consists of kinases and phosphorylation GO terms which were the most significantly overrepresented in infected sample upregulated genes. In total, 352 upregulated kinases were highlighted in this analysis. Mitogen-activated (MAP) kinases direct cellular responses to a large array of stimuli. Here, 27 MAP kinases were found to be upregulated, including some known key players in innate immune response: ANP2, MKK2, MPK8 and MPK3. Yet, seven calcium/calmodulin-dependent protein kinases (CDPKs), which may trigger the plant immunity upon cell calcium influx, were found to be upregulated (Table S9).

Besides these MAP kinases, many groups of kinases showing homologies between each other and with known or supposed PRRs were also found to be upregulated in infected plants, including 53 lectin receptor-like kinases (45 G-type and 8 L-type), including 5 LECRK-like kinases involved in innate immunity [41], 23 LRK10-like homologous to a receptor-like protein kinase involved in wheat rust resistance [42], 12 RLKs homologous to Arabidopsis gene At3g47570 showing similarity to the gene RXam1 conferring resistance against a strain of *Xanthomonas axonopodis* pv. *manihotis* [43], three homologs of FLS2 known as the PRR that determines the specific perception of the bacterial flagellin flg22 [44], one EFR known as PRR interacting with the peptide elf18 (derived from bacterial EF-Tu) [45], and one homolog of Hcr9-4E providing resistance against *Cladosporium fulvum* [46]. Beside immunity-related kinases, 21 kinases showing homology with RLP12-like proteins involved in meristem maintenance were also upregulated in infected plants (Table S9).

#### 3.3.2. Disease Resistance and PR Genes

The majority of disease-resistance genes in plants encode nucleotide-binding site leucine-rich repeat (NBS-LRR) proteins. In the present study, 63 genes annotated in ITAG3.2 CDS as either disease resistance proteins and/or NBS-LRR, TIR-NBS-LRR, and CC-NBS-LRR were found to be significantly upregulated in infected plants. Among these genes, 20 putative late blight resistance R1, 15 TMV resistance protein N-like, four disease resistance RPP-like (for resistance to *Peronospora parasitica*), three resistance gene analogs (RGA), two disease resistance protein RPM1 and one disease resistance protein RPS2 conferring resistance to *Pseudomonas syringae*, were found. Beside NBS-LRR, an extracellular leucine-rich repeat (eLRR) gene, Ve2, responsible for verticillium wilt disease resistance, was also found among the upregulated genes (Table S10).

Because the expression of PR proteins is a marker of SAR induction, upregulated genes were checked for the presence of these proteins. This analysis showed that members of 16 families of PRs [47] were present among significantly upregulated genes. A summary of these genes is presented in Table 1 and mentioned in detail in Table S11.

#### 3.3.3. Transcriptions Factors (TFs)

Ontology enrichment showed that the GO term “DNA binding transcription factor activity” was significantly overrepresented in upregulated genes. In total, 249 genes annotated as TFs were found to be significantly upregulated, including 37 putative WRKY known as elicitor-responsive transcription factors, 22 MYB related, 21 NAC domain-containing proteins, 18 ethylene-responsive, 10 heat shock factors, six MADS-box proteins, six nuclear TFs Y, four LOB domain-containing proteins, four bZIP among which two BZIP17, and a BZIP60 involved in salt and osmotic stress responses and in the activation of unfolded protein response (UPR) respectively, three TGA (TGA2.1, TGA2.3 and TGA4), two pathogenesis-related genes transcriptional activators (PTI5 and PTI6) which activate the defense genes of plants, two ethylene-insensitive 3, 2 MYC (MYC2, MYC3) involved in jasmonic acid (JA) signaling pathways, and a DELLA protein GAI which probably represses the gibberellin (GA) signaling pathway. Remarkably, the key regulator of salicylic acid (SA)-mediated gene expression in systemic acquired resistance, NPR1 protein, was also found among the upregulated genes (Table S12).

Conversely, 153 putative TFs were found to be significantly downregulated. A large diversity was represented among these: six sigma factors (sig A, B, C, D and F) known to regulate the specific transcription of chloroplast related genes, several TFs involved in development and/or floral induction: four auxin response factors, three scarecrow-like proteins, three squamosa promoter-binding-like proteins. Like for upregulated TFs, many uncharacterized TFs were found (Table S12).

#### 3.3.4. Oxidation-Reduction Process

Ontology enrichment revealed many genes involved in oxidation-reduction processes, including 83 cytochromes P450 primarily involved in the formation of terpenoids (Table S13), 23 peroxidases already cited as PR-9 were present and 4 prolyl 4-hydroxylases (P4H4, P4H6, P4H9 and P4H10) which catalyze the post-translational formation of 4-hydroxyproline in -Xaa-Pro-Gly-sequences in proline-rich protein like the plant cell wall glycoproteins: extensins, hydroxyproline-rich glycoproteins, lectins and arabinogalactan proteins, and 13 aminocyclopropane carboxylate oxidases involved in the synthesis of ethylene.

#### 3.3.5. Protein Ubiquitination

In this study, 36 E3 ubiquitin-protein ligases involved in ubiquitination were found to be significantly overexpressed in infected plants (Table S14). This can reveal the polyubiquitination of proteins and their targeting for destruction by the proteasome. However, it can also be linked to the modification of protein’s activity, interactions, or localization. Remarkably, a Avr9/Cf-9 rapidly-elicited protein 1 induced by *Cladosporium fulvum* was found among these ubiquitin-protein ligases.

#### 3.3.6. DNA Methylation and Chromatin Remodeling

Highlighted by the nucleosome GO term which was overrepresented in the upregulated genes (*P* = 0.0001), 27 histone genes (4 H1, 4 H2A, 4 H2B, 10 H3 and 5 H4) were found to be overexpressed in infected samples indicating a significant chromatin remodeling. Likewise, the two core subunits of the condensing complex (SMC2 and 4) were upregulated in infected plants. When it comes to methylation, DRM2, a cytosine methyltransferase involved in de novo methylation in a sequence-independent manner [51], was found to be strongly overexpressed (LogFC = 4).

Conversely, a chromatin remodeling protein EBS-like supposedly involved in preventing histone acetylation through methylated histone binding and 2 DNA (cytosine-5)-methyltransferases (CMT3 and 1B-like) were found to be downregulated (CMT3 being a methyltransferase that is required for the maintenance of DNA methylation at CHG sites [52]).

The situation regarding the enzymes involved in histone modifications was difficult to clarify, since several histone acetyltransferases, deacetylases, and histone-lysine N-methyltransferases were found to be both up and downregulated. Moreover, a BLASTX analysis of several of those genes did not allow us to confirm their annotation in ITAG3.2 release (Table S15).

#### 3.3.7. RNAi

The RNA silencing machinery was also highlighted in this gene expression analysis, and in order to understand how this machinery is impacted during CEVd infection, all genes and microRNA (miRNA) precursors were isolated from the list of differentially-expressed RNA ends and small RNAs (Table 2).

The analysis of Dicer-like protein (DCL) in infected plants showed that (i) according to PARE sequencing, DCL 2b was upregulated in infected plants; while the expression of DCL 2d was activated, (ii) DCL 2a, 2b and 2d were also significantly degraded into small RNAs. The mapping pattern of siRNA on the latter three DCL2 mRNAs indicated RNAi degradation through the production of phasiRNAs, which was confirmed for DCL 2a and 2b using UNITAS. Carefully checking of mappings allowed us to rule out the involvement of DCL 2c, to which some PARE and small RNA reads mapped due to sequence similarities shared between the four DCL 2 paralogs. Furthermore, the analysis of RNA end sequences also showed that DCL 4 was significantly upregulated in infected plants, and DCL1 and DCL3 were expressed in both mock and infected plants, but no difference of expression could be found (Table S16).

When it comes to argonautes, only argonaute 2a was found to be both significantly upregulated and degraded through phasiRNA production in infected plants. Argonaute 4a, known to be involved in DNA methylation, was also found to be upregulated. Mapping quality check allowed us to rule out the expression of argonaute 2b and 3 in this study. Argonautes 1, 5, 6, 7, and 10 were found to be present but not differentially expressed between mock and infected plants (Table S17).

RNA-dependent RNA polymerases (RdRP) were also checked for differential expression. Among the five expressed RdRP (2 RdRP2, 1 RdRP5 and 2 RdRP6), only one RdRP2 was found to be upregulated (Table S18).

Several miRNAs/precursors miRNAs were also found to be upregulated according to PARE quantification, and differential quantification of small RNAs highlighted that miR4376, which is known to regulate the expression of an autoinhibited Ca(2+)-ATPase [53], a potential key player in innate immunity, was significantly overrepresented in infected samples.

It is worth noting that several genes involved in RNAi, SAR, and innate immunity, presented a higher number of both small RNAs and RNA ends in infected samples, indicating a potential control of these genes through RNAi (Table S19). However, except for a leucine-rich-repeat receptor-like protein (Solyc12g009520.2.1), DCL 2b, DCL 2d, and argonaute 2a, the scattered distribution of mapped small RNAs on most of these genes, as well as the size of these small RNAs (often greater than 24-nt), were not compatible with an RNAi origin. Conversely, the four identified genes showed a sRNA distribution consisting of a series of peaks, which is compatible with RNAi degradation in infected plants, and was confirmed for three genes as phasiRNAs using UNITAS.

#### 3.3.8. Translation

Many GO terms enriched in the downregulated genes showed that the translation machinery is highly-affected by CEVd infection. Among the genes concerned, 54 ribosomal proteins (35 chloroplastics), 23 tRNA-synthases and methyltransferases, 7 elongation factors, 3 translation initiation factors (2 chloroplastic), and 2 chloroplastic peptide chain release factors, were downregulated (Table S20).

Pentatricopeptide repeat-containing proteins (PPR) bind RNA in a sequence-specific and influence processing, splicing, editing, stability, and translation of RNAs. In this study, 113 PPRs were found to be downregulated in infected plants in which 25 were associated with mitochondrion and 37 with chloroplast. Conversely, 11 putative PPRs were upregulated in infected plants (Table S21).

#### 3.3.9. Photosynthesis

Several genes related to photosynthesis and included in different GO terms were significantly downregulated in infected plants. All major protein complexes involved in photosynthesis were impacted: the light-harvesting complexes were affected with the down regulation of 32 chlorophyll a/b-binding proteins as well as 4 proteins involved in chlorophyll synthesis, 38 proteins associated with photosystems I and II were downregulated, 2 proteins involved in the NDH complex as well as 7 subunits of the chloroplastic ATP synthase, and a subunit of the cytochrome b6-f complex, were also downregulated (Table S22).

#### 3.3.10. Membrane and Excreted Proteins

Membrane-related GO terms were significantly overrepresented in both up and downregulated genes, indicating a profound change in cell and organelle membranes. Besides the reported changes occurring in the chloroplastic membranes, a diverse array of membrane proteins was found to be downregulated in infected plants, including 19 NRT1/ PTR FAMILY proteins involved primarily in nitrate and peptide transport, 13 proteins detoxification, 6 PIN and 6 WAT1-related proteins involved in auxin transport and homeostasis, 11 aquaporin-like proteins which facilitate the transport of water across cell membrane, and 11 sugar transporters (Table S23).

When it comes to upregulated membrane proteins, many membrane proteins were also highlighted, including 13 ABC transporters (4 being pumps for glutathione S-conjugates), 6 copper transporters, as well as 7 purine permeases (Table S23).

Besides membrane proteins, excreted proteins were also highlighted in upregulated genes in the ontology enrichment. Along with the aforementioned PR genes, several genes also known to be involved in biotic stresses have been found: six phytosulfokines, peptide hormones which trigger cellular dedifferentiation and redifferentiation upon binding to their membrane receptor, four expansin-like proteins which induce wall stress relaxation, two metalloendoproteinases which may play a role in the degradation and remodeling of the extracellular matrix in response to stresses, and two serpin-like proteins which are potent inhibitors of mammalian chymotrypsin-like serine proteases (Table S24).

#### 3.3.11. Hormone Related Genes

Because for each studied hormone, related genes were found to be both up and downregulated, the relative numbers of up and downregulated genes were compared through binomial regression in order to identify the general trends (Tables S25 and S26). This analysis showed that for four hormones related to plant defense, i.e., salicylic acid, abscisic acid, ethylene, and jasmonic acid, the proportions of upregulated genes was highly significantly different from the proportion of downregulated genes (*P* < 0.0001). Conversely, for cytokine, relatively more downregulated genes were found than their upregulated counterparts (*P* = 0.05). No significant differences were found for auxin, brassinosteroid and gibberellin related genes.

## 4. Discussion

This study evidenced CEVd degradation, tomato PTGS targets of CEVd, and tomato transcriptome response upon CEVd infection. *In silico* analysis of potential pospiviroid PTGS targets allowed us to further confirm and extrapolate the experimental results to other pospiviroids.

In terms of methodology, although PARE read quantification is not generally used to identify differentially-expressed genes, the information provided in this publication was supported by the fact that (i) most of the degradome is not the result of small RNA-mediated cleavage [54], (ii) the global shape of gene read maps were very similar in infected and mock samples in hundreds of visually inspected genes, (iii) an exhaustive check of CEVd triggered PTGS sites was performed visually by mapping, and (iv) results of this analysis strongly correlated with previous studies and symptoms development (see here after), as well as the already demonstrated SAR activation [3].

### 4.1. CEVd Degradation

**Figure 5:**
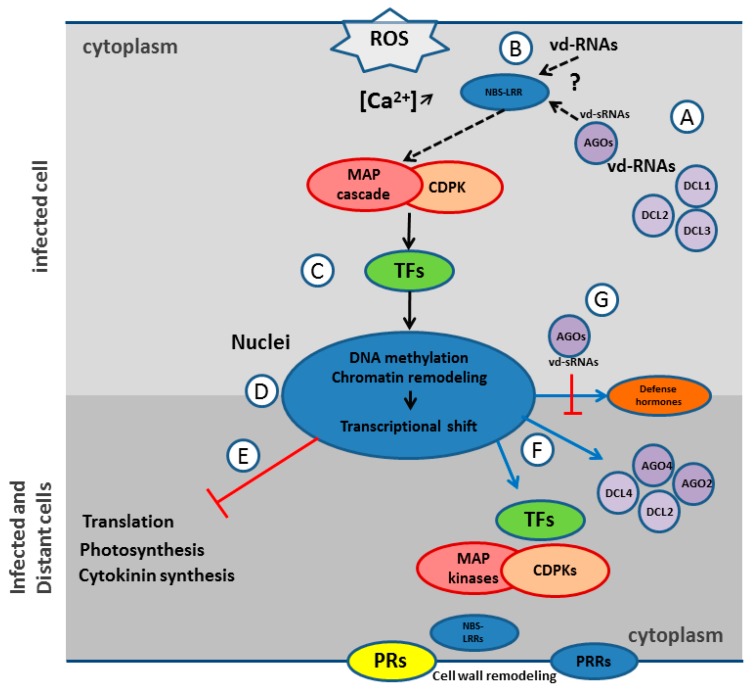
Hypothetical and simplified model of innate immunity and SAR activation by pospiviroids: (**A**) In infected cells, the viroid derived RNAs (vd-RNAs) are degraded by Dicer-like (DCL) 1, 2, and 3 into viroid-derived small RNAs (vd-sRNAs) which are loaded in argonautes (AGOs) to further degrade vd-RNAs. (**B**) vd-RNAs or AGOs loaded with vd-sRNAs are recognized directly or indirectly by nucleotide-binding site leucine-rich repeat (NBS-LRR) proteins which trigger effector-triggered immunity (ETI). (**C**) After reactive oxygen species (ROS) production and Ca^2+^ cytoplasmic influx, MAP kinase cascade (MAP cascade) and calcium-dependent protein kinases (CDPK) activate transcription factors (TFs), which transduce the innate immune signal. (**D**) Host DNA undergoes methylation and chromatin remodeling which lead to a massive transcriptional shift. (**E**) This shift induces a repression of translation, photosynthesis, and cytokinin biosynthesis, which leads to symptoms. (**F**) This shift also induces the systemic activation of immunity defenses through the overexpression of innate immunity related proteins ranging from pathogen sensors, e.g., pattern recognition receptors (PRRs) and NBS-LRRs, to signal transductors, e.g., MAP kinases, CDPKs, TFs, and defense hormones, and eventually, to defense proteins, e.g., RNAi machinery, PR genes, and cell wall remodeling proteins. (**G**) As counter-defense, pospiviroids contain specific sequences in their genome which, when present in vd-sRNAs and loaded in AGOs, trigger the PTGS degradation of mRNAs involved in dedicated defense.

The strand polarities of vd-sRNAs found in this study showed that these latter derived primarily from the positive strand of CEVd. The mappings of these vd-sRNAs on CEVd genome also showed the characteristic patterns consisting of variable peaks and valleys along each strand as observed for different viroids [15,55,56]. The distribution of vd-sRNA lengths, along with the expression of the different DCLs, indicate that DCL1, DCL2s, DCL3, and DCL4 could all be involved in the degradation of viroidal RNAs. However, while the overexpression and activation of DCL2b and 2d respectively can clearly be linked to the high amount of 22-nt vd-sRNAs observed, the overexpression of DCL4 is difficult to assign to 21-nt vd-sRNA production, since it could also be involved in the enhanced phasiRNA degradation observed in the infected samples, as well as in RdDM [57]. Since DCL4 is thought to need long, near-perfect dsRNA as substrate [58], and as almost no symmetry was found between positive and negative strands in terms of 21-nt vd-sRNAs indicating imperfect dsRNA origin, the data suggests that these latter sRNAs are mainly produced by DCL1 (Figure 5A). This would also explain the divergent role of DCL4 observed between RNA viruses which are negatively affected by DCL4 compared to PSTVd whose concentration decreases with that of DCL4 [11]. Perfect long ds-RNA structures of virus replicative intermediates would be targeted by DCL4, while viroids would remain protected from DCL4 degradation thanks to their imperfect ds-RNA structures.

In agreement with this hypothesis, the analysis of RNA ends confirmed the presence of a significant number of predominant subgenomic copies of CEVd, as demonstrated by Minoia et al. [59]. This suggests the involvement of these CEVd genomic fragments, mainly from the positive strand, in the formation of dicer recognizable imperfect ds-RNAs. These imperfect ds-RNA degradations would also explain the presence of a significant amount of vd-sRNAs of unexpected sizes (18 to 20-nt and 23-nt long), which follow similar mapping patterns of what would be their canonical, 21, 22, or 24-nt duplex counterparts (Figures S1 and S2).

It is also worth noting that the polarity ratio discrepancy observed between vd-sRNAs and RNA ends indicate that positive copies of CEVd are more prone to DCL degradation, while the negative copies are more targeted by RISC. The RISC degradation of negative strand being supported by hot spots of 5′ extremity of RNA ends located where peaks of complementary vd-sRNAs can be observed and the fact that AGO2A is both upregulated in infected samples and targeted by the viroid. This evidence and hypothesis thus supports a complex process of viroid RNAi degradation which is compatible with both the findings of [60] that PSTVd titer decreases with the overexpression of AGO1, AGO2, AGO4, and AGO5, and the model developed in [11], where RISC degradation of viroids allows us to understand why PSTVd titer increases when DCL4 is suppressed. They also allow us to understand why RNAi degradation of CEVd, and more broadly, pospiviroids, leads to asymmetric profiles of vd-sRNAs between positive and negative strands, contrary to what its presumed action on perfect ds-RNA would suggest.

### 4.2. Silencing Targets of CEVd

Out of the five PTGS sites highlighted in this study, three could directly modulate the fitness of CEVd variants and/or the symptoms caused by the severe isolate used here.

The mRNA of the translational activator gcn1, partially silenced by CEVd, is known to be involved in general protein synthesis repression and increased translation of specific mRNAs through its interaction with eukaryotic initiation factor 2 alpha kinase Gcn2 (eIF2-α kinase Gcn2) in response to stress. Considering that GO term enrichment suggests that protein synthesis was found to be dramatically repressed by CEVd, it could be speculated that this repression is controlled by this translational activator, which stimulates the eIF2-alpha phosphorylation mediated by GCN2 kinase. Interestingly, eIF2-alpha is a central player in the innate response to infection in mammals, and many viruses have evolved strategies to avoid its phosphorylation, which triggers the repression of translation initiation [61]. Moreover, eIF2-alpha is also the substrate of the PKR which is activated upon double-stranded viral RNAs binding in mammalian. It should be noted, however, that no pPKR has been found so far [27], and that GCN2 does not seem to respond to viral infection in plants [62].

When it comes to the nucleolar protein 12, it could be hypothesized that the PTGS inactivation of this RNA-binding protein would either slow down the detection of the viroid by the plant defense or avoid the hindrance of essential pospiviroid sites by this undesired plant protein.

The potential roles of AGO2a, whose mRNA is degraded through phasiRNA production triggered by vd-sRNAs, is developed above and in the following paragraphs.

Concerning the involvement of vd-sRNAs in RdDM, *in silico* analysis showed that the promoter region of eight downregulated genes showed similarities with vd-sRNAs. Many genes also presented similarity with vd-sRNAs in their introns and exons, possibly leading to DNA methylation. Furthermore, proteins involved in de novo DNA methylation, i.e., DRM2, DCL2, DCL4, and AGO4, were found to be upregulated in infected samples. However, although 21–22 nt sRNAs could presumably trigger PolV-RdDM, it is worth noting that none of the CEVd-derived sRNAs highlighted in this study spanned the canonical 24 nt length required for PolIV-RdDM [56]. Moreover, further analyses would be needed to confirm whether these DNA targets would be specifically methylated independently of the global methylation changes observed in plants facing biotic stresses [57].

*In silico* analysis of potential silencing sites of pospiviroid derived sRNAs in tomato showed striking overrepresentation of some gene ontology terms, indicating that the nucleotide sequences of these non-coding RNAs are not only under a selection pressure to maintain their conformation involved in protein interaction, but also to allow the downregulation of specific host proteins through silencing. Remarkably, this analysis showed highly significant overrepresentation of cytoskeleton related terms which highlighted several kinesin genes. Although plant viruses depend on cytoskeleton for their intracellular transport, little is known about viroid-cytoskeleton interaction. However, it is worth noting that when the 4/1 protein, showing similarity with kinesin but absent in tomato, was downregulated by silencing in tobacco, increased long distance movement of PSTVd was observed [63]. Yet, the microtubule network, to which kinesins bind, is known to be involved in immunity response by delivering cell wall components and potentially also other defense agents. Moreover, several effectors alter the function of microtubule-associated proteins during the immune response [64]. Interestingly, β-1,3-glucan biosynthesis, which is related to callose deposition between the cell wall and the plasma membrane to limit the penetration of pathogens and the cell-to-cell movement of viruses and viroids [65], was also highlighted in overrepresented ontology terms. Gene ontology enrichment also revealed potential PTGS sites in many genes related to innate immunity and RNAi. Remarkably, disease resistance proteins able to trigger ETI were found to be among the most stable PTGS duplexes and the most conserved pospiviroid genomic regions.

### 4.3. Innate Immunity Activation

The transcriptome analysis showed that tomato plants react to CEVd by a major gene expression shift supposedly governed by kinases, transcription factors, chromatin remodeling, and DNA methylation (Figure 5). The consequences of this shift are the downregulation of genes involved in photosynthesis and in protein synthesis and the massive overexpression of a large array of genes involved in innate immunity. The CEVd activation of genes like MAP kinases, CDPKs, defense related TFs, and members of 16 PR families, is thus fully compatible with the induction of the innate immunity proposed by Zheng et al. [4] and with the results obtained in several other studies looking for PSTVd impacts on the tomato transcriptome [66,67] and metabolism [68].

This work also confirmed the activation of SAR observed by López-Gresa et al. [3], which is probably linked to the systemic overexpression of the complete arsenal of innate immunity ranging from pathogen sensors (PRR and NBS-LRR), to signal transduction (MAP kinases, CDPKs, TFs, defense hormones), and eventually, to defense proteins (RNAi machinery, PR genes, cell wall remodeling proteins) (Figure 5F). Indeed, it could be speculated that this general reinforcement of cell defenses makes the plants more prompt to react to further infection, whatever the pathogen and PRs are well known as SAR markers. These results, together with previous results [4,66,67], thus showed that pospiviroids are able to consistently activate the innate immunity and SAR of tomato, even in the late stage of infection (47 dpi here), but that this arsenal fails to curb these pathogens.

How tomato activates its innate immunity in response to CEVd is not known, but the proteins highlighted through the mapping of artificial pospiviroid vd-sRNAs and RNAi components are good candidates for both the recognition of vd-RNAs and the activation of innate immunity and SAR. The overexpression of DCL2s and DCL4 would suggest a predominant role of RNA silencing machinery, and it could be hypothesized that a protein-protein interaction with DCL2 and/or the silencing of essential regulating genes by DCL2 derived phasiRNAs are the initial triggers upon pathogenic dsRNA infection. Remarkably, BLASTX analyses of mammal ds-RNA receptors RIG-1 and MDA5, using tomato genome as a reference, revealed similarities with DCL3 and DCL4 respectively. When it comes to the possible activation of the innate immunity by DCL2 phasiRNAs, it should be noted that a stringent mapping of these phasiRNAs on tomato CDS conducted in this study did not reveal any good candidates for immunity activators. Interestingly, although the mechanism is still unknown, AGO2 has been found to play an essential role in antiviral defense [69,70]. Remarkably, ago2 mutants showed increased symptoms but no impact on the viral accumulation. In the present study, the fact that this severe isolate of CEVd triggers both the upregulation and the degradation of AGO2A suggests that AGO2 also plays a pivotal role in antiviroidal defense and modulation of symptoms. The recognition of vd-sRNA loaded AGO2A by innate immunity receptors could also be an interesting avenue of future research (Figure 5B).

Recently, ds-RNAs-triggered immunity operating independently of RNA silencing in plant was also demonstrated [5]. However, the specific dsRNA receptor was not identified. Considering these findings, together with the absence of pPKR, the TIR-NBS-LRR and the NB-LRR proteins identified by *in silico* analysis as common potential PTGS target of pospiviroids would constitute good RNA receptor candidates for the direct or indirect recognition of vd-RNAs and the induction of ETI and SAR (Figure 5B). Unfortunately, too few PARE reads were available at the corresponding PTGS sites to confirm the importance of these proteins. Another explanation for these striking common potential immunity sensor targets would be the inactivation of plant immunity for viruses able to allow the transmission of pospiviroids in their natural hosts by trans-encapsidation.

When it comes to symptom induction, the long-lasting impediment of photosynthesis and protein synthesis, along with the potential protein degradation activity, certainly impacts the growth and the phenotype of CEVd infected plants. Yet, the overall downregulation of genes involved in cytokinin production/signaling could also explain the reduction of the apical meristem dominance in pospiviroid infected plants. Since cytokinin is an important plant growth regulator involved in cell division and differentiation, the typical dwarfing symptom and epinastic leaves induced by pospiviroids could be linked to the phenotypes observed in tobacco showing reduced concentrations of cytokinin [71] (Figure 5E).

Moreover, the drastic impact of pospiviroid on all complexes of the photosynthesis machinery, especially chlorophyll biosynthesis and chlorophyll binding proteins, should have a link with the typical chlorosis symptoms observed in the top leaves of pospiviroid infected tomatoes, and was particularly visible with the CEVd strain used in the two tested tomato varieties (cv. Minibel and cv. Heinz 1706). As innate immunity activation is associated with the production of reactive oxygen species (ROS), it could also be hypothesized that the appearance of necrosis observed in leaf vascular tissue is due to a continuous oxidative stress [72].

To explain how pospiviroid strains modulate symptoms, several variables probably come into play, among which the most important are probably the efficiency of viroid replication and trafficking, viroid degradation, and the silencing of plant targets.

So far, only the RNAi is known to constitute an effective defense against viroids. However, RNAi can also have a deleterious effect on the plant, since its action can lead to undesired silencing of essential genes. In the present study, the degradation of AGO2A possibly leads to a better viroid replication, and thus, to increased symptoms. The gene silencing through PTGS highlighted in this study would also explain, at least in part, the additional alteration of the host phenotype induced by the severe strain of CEVd used.

## 5. Conclusions

This work confirmed, at the transcriptome level, the activation of innate immunity and SAR by pospiviroids. The downregulation of photosynthesis, translation, and cytokinin-related genes in infected plants provided a good explanation of the symptoms caused by pospiviroids in tomato. The data generated suggests a joint degradation of CEVd by DCLs and RISC from subgenomic copies. The results also confirmed the key role of AGO2 which is both upregulated and degraded in samples infected with the severe strain of CEVd used. *In silico* analysis of potential PTGS sites indicated that evolution shaped pospiviroid sequences, not only for conformation maintenance, but also to target the silencing of specific host genes. Based on this computer analysis, and considering the experimental results, potential innate immunity receptors recognizing viroid-derived RNAs were suggested, allowing us to propose a first hypothetical model of ETI and SAR activation by pospiviroids.

## Figures and Tables

**Table 1:** Significantly upregulated PR genes (LogFC > 1 and FDR < 0.05) in infected plants clustered according to their PR family, supposed functions [47,48,49,50] and mean overexpression (log FC).

PR Family	Upregulated Genes	Supposed Functions	Mean Overexpression
1	13 genes related to PR-1	Multiple roles ranging from antimicrobial function and defense signal amplification to potential sterol or effector recognition	6.5
2	12 glucanases	Modulators of callose and salicylic acid-dependent defense responses	5.1
3, 4, 8 and 11	18 chitinases	Antifungal properties	5.4
5	5 thaumatins, 3 osmotins and 1 PT-5x	Membrane permeabilizing functions	5.8
6	23 proteinase inhibitors (PIs)	Proteinase inhibitors	4.6
7	16 subtilisin-like proteases and 3 P69 proteins	Proteinases	3.6
9	23 peroxidases	Peroxidases	3.7
10	a S-norcoclaurine synthase 2-like a TSI-1 (tomato stress induced-1)	Antimicrobial activity and in vitro ribonuclease activity, enzymatic activities in plant secondary metabolisms, ligand-binding ability	8.3
12	3 defensin-like proteins	Membrane permeabilizing functions	5.7
13	a thionin-like protein	Membrane permeabilizing functions	2.3
14	9 lipid transfer proteins	Membrane permeabilizing functions	4.8
15	4 germins	Multiple enzymatic, structural and receptor functions	7.3
16	5 germin-like proteins	Multiple enzymatic, structural and receptor functions	2.6

**Table 2:** Overexpression of the RNAi machinery components in CEVd infected tomato plants.

Tomato Gene ID	RNAi Machinery Components	PARE		Small RNA		Number of phasiRNA Detected
		Log FC	FDR	Log FC	FDR	
Solyc06g048960.3.1 ^a^	Dicer-like 2a	-	-	2.2	0.002	44
Solyc11g008540.2.1 ^a^	Dicer-like 2b	3.0	4.80e−5	4.2	5.27e−11	53
Solyc11g008530.2.1 ^b^	Dicer-like 2d	6.2	8.92e−6	5.2	3.09e−12	-
Solyc07g005030.3.1 ^a^	Dicer-like 4	1.6	0.0008	-	-	-
Solyc02g069260.3.1 ^a^	Argonaute 2a	2.7	0.0002	11.1	3.50e−24	102
Solyc01g008960.3.1 ^a^	Argonaute 4a	1.3	0.0003	-	-	-
Solyc03g114140.3.1 ^a^	RNA-dependent RNA polymerase2	1.18	0.0007	-	-	-
NR_108010	MIR4376	-	-	3.2	1.86e−8	
NR_108011	MIR6022	1.3	0.04	1.80	0.006	
NR_130104	MIR171e	-	-	2.50	0.018	
LM611859	MIR403	-	-	2.3	0.04	
NR_130094	MIR9474	1.5	0.002	-	-	
NR_108004	MIR5300	5.7	0.009	-	-	
LM611867	MIR9472	5.5	0.012	-	-	

^a^ PARE reads present in both infected and mock plants; ^b^ PARE reads only present in infected plants.

## Supplementary Materials

The following are available online at http://www.mdpi.com/1999-4915/10/11/587/s1. Figure S1: vd-sRNA maps on the positive strand of CEVd genome for each sRNA length, Figure S2: vd-sRNA maps on the negative strand of CEVd genome for each sRNA length, Figure S3: PARE reads maps on positive and negative strands of CEVd genome, Table S1: Summary of sequencing libraries, Table S2a: Potential targets of artificial vd-sRNAs of pospiviroids, Table S2b: Gene ontology enrichment of potential PTGS targets of artificial vd-sRNAs, Table S3: Potential RdDM targets of CEVd derived sRNAs in promoter regions of tomato, Table S4a: PTGS site of CEVd derived sRNAs, Table S4b: Normalized count of PARE 5′ ends for the five PTGS targets of vd-sRNAs, Table S5: up and downregulated genes according to PARE reads quantification, Table S6: Genes for which different amounts of mapped small RNA reads were found in infected compared to mock samples, Table S7: Gene ontology enrichment in genes overexpressed in infected samples, Table S8: Gene ontology enrichment in genes downregulated in infected samples, Table S9: Differentially expressed kinase related genes, Table S10: Differentially expressed disease resistance genes, Table S11: Differentially expressed PR encoding genes, Table S12: Differentially expressed transcription factor encoding genes, Table S13: Differentially expressed cytochrome P450 genes, Table S14: Differentially expressed E3 ubiquitin-protein ligases genes, Table S15: Differentially expressed chromatin related genes, Table S16: Expression of Dicer-like related genes, Table S17: Expression of argonaute related genes, Table S18: Expression of RNA-dependent RNA polymerase related genes, Table S19: Disease related genes upregulated in infected plants for both PARE and small RNA reads, Table S20: Downregulated genes related to translation, Table S21: Differentially expressed pentatricopeptide genes, Table S22: Downregulated genes related to photosynthesis, Table S23: Differentially expressed genes related to membrane proteins, Table S24: Upregulated excreted protein genes, Table S25: Expression of hormone related genes, Table S26: Expression of hormone related genes statistical analysis.
