# Supplementary material for: Innate Immunity Activation and RNAi Interplay in Citrus Exocortis Viroid—Tomato Pathosystem
Source: Viruses. 2018 Oct 26;10(11):587. doi: 10.3390/v10110587 (PMC6266551; doi:10.3390/v10110587)

Figure S2 Mappings of vd-sRNAs derived from the negative strand of CEVd for each of the vd-sRNA length ranging from 18 to 24 nt and for both biological replicates

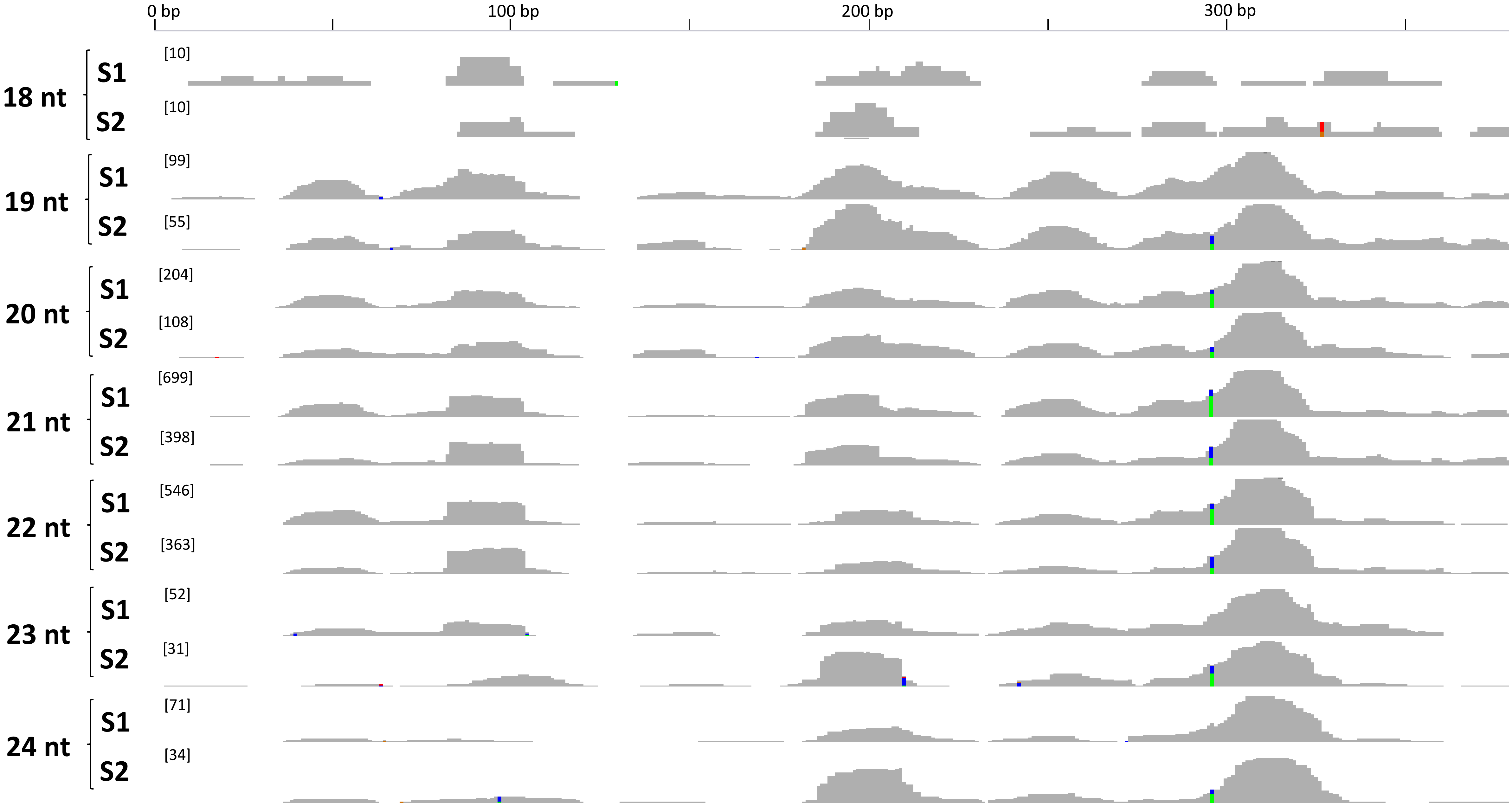

Supplement: Supplementary file 1 [file viruses-10-00587-s001.zip › viruses-354845-SI/Figure_S2.pdf]
